# Contributions for the Sanitary Commission Volume

**Published:** 1866-10

**Authors:** 


*Contributions for the Medical Volume to be Published under the Direction of the Sanitary Commission.*—Our readers are doubtless aware that a series of volumes is to be published under the direction of the Sanitary Commission; the different volumes to be devoted respectively to a History of the Commission, to Hospitals, to Military Hygeine, to Surgery, and to Medicine. We are requested by the editor of the volume on Medicine to invite any who served as surgeons or physicians during the war, either at the North or South, to contribute articles relating to camp diseases. All articles received will be carefully examined, and if incorporated in the work, due acknowledgment will be made to the authors. Articles relating to any of the diseases which prevailed in the armies of the United States or among the Confederate troops, will be gladly received. As the volume will be largely circulated, it will be a desirable medium for the diffusion of important facts and conclusions, based on the experience of those who held medical positions during the war. Articles should be sent, if possible, by the middle of September, or, at the furthest, by the first of October. They may be directed to the Medical Committee of the Sanitary Commission, No. 21 West 12th Street, New York City. Editors of Medical Journals are respectfully requested to insert this notice.—*N. Y. Med. Jour.*

---

*Physiological Action of Narceine.*—In the last number of the *Journal de Chimie Medicale* there is an abstract of M. Linne's researches on the above subject, from which we perceive that the following conclusions have been arrived at: (1) Narceine is unquestionably of all the alkaloids of opium that which has the greatest narcotic power. *In the majority of cases* morphia and codeia do not produce as sound or prolonged sleep as results from the use of narceine. (2) Narceine differs from the other alkaloids of opium in producing little perspiration, and in causing no loss of appetite or nausea. (3) So far from producing constipation of bowels, it causes relaxation, and, in large doses, actually gives rise to diarrhœa. (4) It not only produces sleep, but diminishes pain. (5) It has one peculiar action; it suppresses the flow of urine. For this reason M. Linne thinks it might be advantageously employed in cases of nocturnal incontinence of urine amongst children. But it seems to us that, until its action can be shown to be on the bladder rather than on the kidneys, its employment in such cases would be highly improper.—*Lancet.*

